# Anterior endoscopic transcortical approach to a pineal region cavernous hemangioma

**DOI:** 10.3171/2021.4.FOCVID215

**Published:** 2021-07-01

**Authors:** Jiuhong Li, Jiaojiang He, Lunxin Liu, Liangxue Zhou

**Affiliations:** Department of Neurosurgery, West China Hospital of Sichuan University, Chengdu, Sichuan Province, People’s Republic of China

**Keywords:** endoscopic resection, pineal region, cavernous hemangioma, anterior transcortical approach, endoscopic third ventriculostomy

## Abstract

A 57-year-old female presented with headache and dizziness for 3 months. Preoperative MRI revealed a lesion located at the pineal region and back side of the third ventricle, accompanied by hydrocephalus. The infratentorial supracerebellar approach may cause visuomotor, acousticomotor, and hearing disturbances. With the patient in a supine position, the authors used a frontal linear incision that was 3 cm anterior to the coronal suture and 2 cm away from the midline and an anterior endoscopic transcortical approach, which could achieve endoscopic third ventriculostomy, alleviating and preventing hydrocephalus due to postoperative adhesion and resection of the lesion at the same time. The pathological diagnosis was cavernous hemangioma.

The video can be found here: https://stream.cadmore.media/r10.3171/2021.4.FOCVID215.

## Transcript

Anterior endoscopic transcortical approach to a pineal region cavernous hemangioma. This patient was a 57-year-old female. Her chief complaint was headache and dizziness for 3 months. MMSE [Mini-Mental State Evaluation] was 18 scores. Other laboratory findings are unremarkable.

### 0:38 Radiological Features

MRI showed the lesion located at the pineal region and back of third ventricle, which leads to obstructive hydrocephalus. The diagnosis was pineal region lesion (cavernous hemangioma most likely), accompanied by obstructive hydrocephalus. For the lesion located at the anterior side of pineal region, there are four approaches to access the lesion backward. Whether it is possible to access the lesion from anterior direction by endoscope? The answer is yes.

### 1:06 The Reason to Choose Anterior Endoscopic Transcortical Approach

The most commonly used approach is infratentorial supracerebellar approach; however, it might result in visuomotor, acousticomotor, and hearing disturbances due to destructing the pineal, superior, and inferior colliculus in this case.^1^ In order to avoid these defects, tonsillouveal transaqueductal approach may be used; however, it may lead to postoperative obstructive hydrocephalus.^2^ So, we decided to use anterior endoscopic transcortical approach to achieve endoscopic third ventriculostomy (ETV) and resection of the aqueduct lesion.

### 1:42 Position and Incision

With a supine position, we use a left frontal linear incision, and the center of the burr hole is 2 cm ahead of Kocher’s point, which is 3 cm anterior to the coronal suture and 2 cm away from the midline. This approach enables us to achieve an ETV and also access the posterior third ventricle at the same time.

This is the procedure of craniotomy. After entering the lateral ventricle, we use coagulator and scissors to enlarge interventricular foramen. We find the floor of third ventricle sinks and is adherent closely to the basilar artery. Then we coagulate and bluntly separate it.

### 2:33 Endoscopic Third Ventriculostomy

Subsequently, we use coagulator and scissors to make ETV and further enlarge the fistula. Special attention should be paid to protect the basilar artery and surrounding vessels. Then we fill the fistula with Gelfoam. After that, we seek the lesion backward. The lesion locates at the backside of the third ventricle. It is grapelike looking and obstructs the midbrain aqueduct.

### 3:37 Resection of the Tumor

Firstly, we coagulate along the surface of the lesion in a circle. We recognize the boundary between the lesion and normal structures carefully. Then we coagulate to shrink the lesion and expand the operating space. We separate along the boundary of the lesion very carefully to protect brain tissue. The direction of separation should always be toward the lesion. The inlet of aqueduct is revealed after displacement of the lesion. Gelfoam is used to protect surrounding brain tissue. The lesion is hard and calcified. We cut and remove the lesion piece by piece. After the operation, the surgical field is clean and without hemorrhage. Finally, we removed all the Gelfoams in the ventricle before the end of the surgery and check the fistula, which is fluent.

### 6:14 Closure

At last, we made closure layer by layer, including suturing the periosteum. Postoperative CT showed hydrocephalus alleviated. Postoperative MRI revealed gross-total resection of the lesion and also demonstrated our operative pathway. Fluent flowing of cerebrospinal fluid was confirmed in CSF film 3 months postoperatively. Pathological findings show vascular tissue of different sizes and calcification, which confirms the diagnosis of a cavernous hemangioma.

The patient had slightly worsened orientation and concentration 2 days after operation and no other early complication occurred. And MMSE score improved 3 months postoperatively. Neurological specialist physical examination showed good recovery of the patient.

### 7:11 Summary

Finally, here is the summary of the operation. First, surgical approach of neuroendoscopy making needs tailoring to the individual lesion characteristics. Second, the correct procedure for the operation is first making a third ventriculostomy and then removing the lesion. Finally, the key point of the surgery is precise and careful anatomical separation and strict protection of surrounding tissues.

## Acknowledgments

The current study was financially supported by the Sichuan Science and Technology Support Program (no. 2017SZ0195) and Program of Health Commission of Sichuan Province (no. 20PJ051). Three of the pictures shown in the video are from Rhoton’s Collection (Rhoton AL: *Cranial Anatomy and Surgical Approaches*. Lippincott Williams & Wilkins; 2003).

## Author Contributions

Primary surgeon: Zhou. Assistant surgeon: He. Editing and drafting the video and abstract: all authors. Critically revising the work: all authors. Reviewed submitted version of the work: all authors. Approved the final version of the work on behalf of all authors: Zhou. Supervision: Zhou, Li, Liu.
